# Opposite Roles for ZEB1 and TMEJ in the Regulation of Breast Cancer Genome Stability

**DOI:** 10.3389/fcell.2021.727429

**Published:** 2021-08-12

**Authors:** Mélanie K. Prodhomme, Sarah Péricart, Roxane M. Pommier, Anne-Pierre Morel, Anne-Cécile Brunac, Camille Franchet, Caroline Moyret-Lalle, Pierre Brousset, Alain Puisieux, Jean-Sébastien Hoffmann, Agnès Tissier

**Affiliations:** ^1^INSERM 1052, CNRS 5286, Centre Léon Bérard, Cancer Research Centre of Lyon, Équipe Labellisée Ligue Contre le Cancer, Université de Lyon, Université Claude Bernard Lyon 1, Lyon, France; ^2^LabEx DEVweCAN, Université de Lyon, Lyon, France; ^3^Laboratoire d’Excellence Toulouse Cancer (TOUCAN), Laboratoire de Pathologie, Institut Universitaire du Cancer-Toulouse, Toulouse, France; ^4^Gilles Thomas Bioinformatics Platform, Centre Léon Bérard, Cancer Research Centre of Lyon, Lyon, France; ^5^Institut Curie, Versailles Saint-Quentin-en-Yvelines University, PSL Research University, Paris, France

**Keywords:** epithelial to mesenchymal transition, DNA Repair, TMEJ, DNA polymerase theta, replicative stress

## Abstract

Breast cancer cells frequently acquire mutations in faithful DNA repair genes, as exemplified by BRCA-deficiency. Moreover, overexpression of an inaccurate DNA repair pathway may also be at the origin of the genetic instability arising during the course of cancer progression. The specific gain in expression of *POLQ*, encoding the error-prone DNA polymerase Theta (POLθ) involved in theta-mediated end joining (TMEJ), is associated with a characteristic mutational signature. To gain insight into the mechanistic regulation of *POLQ* expression, this review briefly presents recent findings on the regulation of *POLQ* in the claudin-low breast tumor subtype, specifically expressing transcription factors involved in epithelial-to-mesenchymal transition (EMT) such as ZEB1 and displaying a paucity in genomic abnormality.

## Introduction

Genetic abnormalities have been largely described as a major hallmark of cancer. Typically, dysfunctional faithful DNA repair is at the origin of the numerous genomic aberrations driving malignant transformation by endowing cells with adaptive and proliferative advantages (Hanahan and Weinberg, 2011).

DNA double-strand break (DSB) repair pathways are generally classified into two categories, namely homologous recombination (HR) and canonical non-homologous end joining (cNHEJ). HR requires 5′ to 3′ end resection, RAD51 loading, strand invasion and DNA synthesis using an intact homologous template. In contrast, cNHEJ does not necessitate a homologous template and is instead dependent on the KU complex, DNA-PKcs, and XRCC4/LIG4. Alternative end joining pathways (Alt-EJ) including microhomology-mediated end joining (MMEJ) has also been described, which in contrast to cNHEJ acts on the 5′ to 3′ resected DSB HR intermediates. Additionally, MMEJ relies on DNA synthesis directed by short tracts of flanking microhomology leading to typical patterns of microhomology-flanked deletions and insertions. The proteins involved in MMEJ include the 5′ to 3′ resection factors MRE11, RAD50, NBN, CtIP, and EXO1 as well as PARP1 and LIG3. However, the most prominent factor associated with MMEJ is the DNA polymerase Theta (POLθ) encoded by the *POLQ* gene. POLθ is a unique multifunctional enzyme with an N-terminal helicase-like domain linked by a central region to a C-terminal A-family DNA polymerase domain (Seki et al., 2003). As a consequence of the major involvement of POLθ, MMEJ has also been termed theta-mediated end joining (TMEJ) (Schimmel et al., 2017, 2019).

Recently, in the context of mammary cancer, high *POLQ* expression was observed in the most genomically unstable breast cancer subgroup containing HR-deficient tumors (Prodhomme et al., 2021). Conversely, in a subgroup distinguished by low genomic instability, a low frequency of TP53 mutations (Morel et al., 2017) and expression of epithelial-to-mesenchymal transition (EMT) features, as well as low *POLQ* expression were detected (Prodhomme et al., 2021). The EMT program, naturally inducing a phenotypic switch during embryonic development or adult tissue homeostasis by the transcriptional repression of epithelial factors, such as E-cadherin (CDH1 gene), may be expressed during tumorigenesis to confer epithelial-to-mesenchymal plasticity to cancer cells, which then acquire stem-like properties (Ye and Weinberg, 2015; Brabletz et al., 2018; Stemmler et al., 2019). Various transcription factors have been shown to orchestrate EMT, named the EMT inducing-transcription factors (EMT-TF), as Zinc finger E-box binding homeobox 1 (ZEB1). ZEB1 is associated with chemo-resistance and radio-resistance properties partly attributed to phosphorylation of ZEB1 by Ataxia-telangiectasia-mutated (ATM) (Zhang et al., 2014) and to the ZEB1 transcriptional activation of ATM (Zhang et al., 2018). ATM is a central regulator of DNA damage response (DDR) signaling which channels DSB repair into the process of HR.

Several aspects of the mechanisms underlying the choice of DNA repair pathway remain unanswered. Numerous studies have shown that individually both DNA damage repair pathways and the EMT process can be hijacked to promote cancer. What if these mechanisms were interconnected during cancer initiation and/or progression? Here, we address the relationship between replication stress generated by tumor initiation and/or progression and TMEJ or EMT features, and how these factors/processes ultimately contribute to genomic stability.

## Subsections:

### Replication Stress, Genomic Instability, and Cancer Progression

Genome stability is compromised by exogenous insults such as chemical carcinogens and ionizing radiation. Endogenously-induced DNA damage generated during the process of chromosome duplication can also affect the stability of the genome. Then, DNA replication forks can be slowed down or stalled by various natural replication barriers, a process referred to as replication stress (RS) (Zeman and Cimprich, 2014; Macheret and Halazonetis, 2015). RS is detected at early stages of tumorigenesis and is generally considered to be the driving force behind cancer progression (Bartkova et al., 2005; Gorgoulis et al., 2005; Negrini et al., 2010). Indeed, oncogene-driven cell proliferation induces a high level of RS, arising notably from the perturbation of replication origin activation and timing as well as increased conflicts between replication and transcription. It results in under-replicated regions and the persistence of stalled and collapsed forks become major sources of chromosome breakage and instability. If two converging replication forks stall with no licensed origin in-between, a double fork stalling event occurs and the replication of this stretch of DNA has a high probability of being compromised. The main consequence of a double fork stalling event is the generation of under-replicated parental DNA (UR-DNA; also called “unreplicated DNA”), which can persist when the cells enter mitosis and lead to chromosomal breaks inheritable by the next generation of cells (Bertolin et al., 2020; Franchet and Hoffmann, 2020). Generally, collapsed forks also lead to DSBs, hence RS is also largely associated with the generation of DSBs, major threats to genome integrity and cell viability. These chromosomal breakages and alterations provide a permanent sub-population of cellular variants upon which selection could act, a proposed driving mechanism for tumor heterogeneity and development of drug resistance. Clonal evolution in cancer can result from the multiple forms of selective pressures that allow some mutant sub-clones to multiply while others become extinct.

While genomic instability is generally associated with poor prognosis, excessive chromosomal instability is deleterious for cell fitness and is correlated with enhanced cancer outcome, arguing in favor of an appropriate threshold in cancer cells for limiting extremely risky RS and DSBs (Sansregret and Swanton, 2017; Maiorano et al., 2021). Therefore, one of the most important features of cancer cells is the need to adapt to severe replicative defects and the ensuing excessive DSBs that are normally incompatible with cell survival. Importantly, several of these adaptive responses currently represent a very active area of research as they are considered to be therapeutically exploitable. First is the ATR-CHK1 checkpoint response which coordinates the stability of arrested forks and fork repair processes, preventing premature entry into mitosis and ensuring the completion of DNA replication (Saldivar et al., 2017). High expression of the genes encoding the checkpoint mediators CHK1, Claspin and Timeless known to stabilize stalled replication forks upon RS and that could counteract excessive RS in cancer cells, was correlated with poor patient survival (David et al., 2016; Bianco et al., 2019). The second adaptive response corresponds to molecular factors including RAD52 of mitotic DNA synthesis (MiDAS), a process that differs from semi-conservative DNA replication in S-phase and which neutralizes potentially lethal chromosome mis-segregation and non-disjunction by restraining the persistence of under-replicated DNA in mitosis (Franchet and Hoffmann, 2020). MiDAS is described as a form of HR-based DNA repair highly prevalent in aneuploid cancer cells, where it counteracts DNA replication stress that arises at “difficult-to-replicate” loci such as common fragile sites (Bergoglio et al., 2013). The third category, which will be developed in the next paragraph, includes the *POLQ* gene encoding POLθ.

### TMEJ Limits Loss of Chromosomal Integrity

**FIGURE 1:**
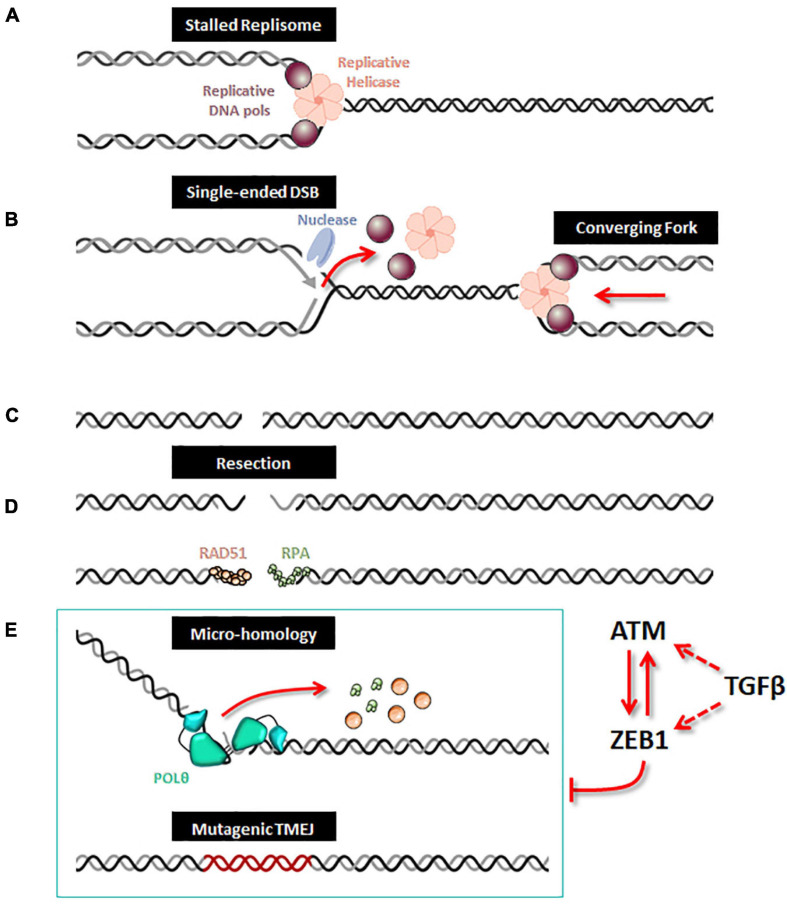
ZEB1/EMT controls TMEJ. When a replication fork is stalled **(A)**, unloading of the replicative helicase and DNA polymerases leads to incision by a DNA nuclease, which creates a single-ended DSB **(B)**. After replication forks completion, a DSB is generated **(C)**. A 5′ to 3′ end resection generates single-stranded DNA, along which RPA and Rad51 loading can occur **(D)**. When homologous recombination is defective, the alternative TMEJ pathway can operate on this resected DSB **(E)**; POLθ is recruited as a dimer which facilitates the proximity of DNA ends and stabilizes synapsed intermediates; the helicase domain of POLθ can displace either RPA or RAD51 and the polymerase domain executes a bidirectional scanning initiated from the 3′ termini to identify internal microhomologies which can be annealed, thus generating 3′ flaps. POLθ then removes the 3′ flaps and starts DNA synthesis with poor processivity and frequent aborted synthesis resulting in a high rate of mutations including deletions and insertions from template switching events. When ZEB1 is expressed and stabilized by ATM, the expression of *POLQ* (encoding the POLθ protein) is decreased and therefore impedes the action of the alternative mutagenic TMEJ pathway on the resected DSB. The TMEJ inhibition by ZEB1 combined with ATM activity enhances accurate homologous recombination. Moreover, ZEB1 activates the transcription of ATM, both being under direct or indirect control of TGFβ signaling.

Although *POLQ* orthologs are present in multiple species (Seki et al., 2003; Seki and Wood, 2008), in normal cells, TMEJ activity for DSB repair is very low and *POLQ* deficiency in several species has been shown to have a minor impact on organismal development (Alexander et al., 2016; Thyme and Schier, 2016). In contrast, in cells that are deficient in HR or NHEJ, including BRCA1/2 mutated cancer cells, POLθ becomes essential, indicative of synthetic lethal genetic interactions between the backup POLθ/TMEJ repair pathway and HR or NHEJ (Ceccaldi et al., 2015; Mateos-Gomez et al., 2015; Feng et al., 2019; Kamp et al., 2020; Carvajal-Garcia et al., 2021; Patel et al., 2021). It has been proposed that POLθ favors end joining of two separated DSBs (distal end joining) (Hwang et al., 2020). Moreover, a study recently revealed a broader landscape of synthetic lethality with POLθ, emphasizing a critical and general role for POLθ in protecting cells from the accumulation of non-productive HR intermediates at sites of DNA replication-associated DSBs, even when canonical DSB repair pathways are functional (Feng et al., 2019), notably TMEJ has been proposed to contribute to the repair of single-ended DSBs at collapsed forks (Wang et al., 2019; Figure 1). Because of its high inaccuracy, TMEJ has been originally considered as a backup DNA repair pathway. However, TMEJ has been proposed to be essential in the repair of collapsed replication forks with sister chromatids containing an inter-strand crosslink (Wyatt et al., 2016; Feng et al., 2019; Schrempf et al., 2021) as well as the repair of G4 quadruplex structures (Koole et al., 2014). Nevertheless, the regulation of TMEJ versus HR need to be further explored for this particular DNA damage. POLθ contains an exonuclease-like domain but lacks 3′→5′ proofreading activity, explaining why POLθ is an error-prone polymerase (Arana et al., 2008). Because of its low fidelity and the unique thumb domain that holds positively charged residues to grasp the unstable primer terminus, POLθ has the ability to extend DNA from mismatched primers (Zahn et al., 2015). In the TMEJ process, microhomologies are identified by a bidirectional progression to a maximum of 15 nucleotides into flanking DNA through a scanning mechanism initiated from the 3′ terminus (Carvajal-Garcia et al., 2020). Aborted synthesis is frequent for POLθ as it is not sufficiently processive, leading to additional rounds of microhomology search, annealing and synthesis which can be observed in some cancer genomic scars (Pettitt et al., 2020), such as insertions of 3 to 30 bp of sequences identical to flanking DNA. Despite these mutagenic features, POLθ/TMEJ has been clearly demonstrated to prevent some chromosome translocations and mis-segregations by fixing DSB *per se*, i.e., limiting loss of chromosomal integrity (Hwang et al., 2020). Hence, it is possible that the high expression of POLθ observed in multiple cancers, frequently defined as a bad prognostic marker (Lemee et al., 2010; Pillaire et al., 2010; Allera-Moreau et al., 2012), has evolved to cope with chromosome fragility and assist the completion of DNA replication to prevent catastrophically large deletions and aberrant chromosome segregation. The increase in mutational frequency as well as short deletions and insertions associated with TMEJ could be the price to pay for cancer cell survival (Figure 1). Several companies are now considering POLθ as a strong therapeutic target and are on the verge of launching POLθ inhibitors, targeting especially breast and ovarian cancers with BRCA1/2 deficiency.

### ZEB1 Controls TMEJ

Initially, indirect evidence highlighted a role for EMT in the regulation of TMEJ. The expression of ZEB1 is activated by the cytokine transforming growth factor-β (TGFβ) signaling pathway (Shirakihara et al., 2007), the inhibition of which compromises the HR and cNHEJ DSB repair mechanisms and increases the reliance on the error prone alt-EJ/TMEJ pathway. TGFβ signaling impediment leads to a significant increase in chromosomal aberrations in irradiated cells from human papilloma virus-positive head and neck squamous cell carcinoma (HNSC) (Liu et al., 2018). More recently, TGFβ was confirmed to broadly control the DNA damage response and to transcriptionally inhibit alt-EJ/TMEJ genes, such as those of *POLQ*, *PARP1*, and *LIG1*. Interestingly, the identified TGFβ and alt-EJ gene signatures were anticorrelated in HNSC, in glioblastoma, squamous cell lung cancer, and serous ovarian cancers. Furthermore, tumors classified as low TGFβ and high alt-EJ were characterized by an insertion-deletion mutation signature containing short microhomologies across several cancers (Liu et al., 2021).

Further insights have been recently gained when ZEB1 was shown to modulates TMEJ activity by directly inhibiting *POLQ* expression (Prodhomme et al., 2021). Essentially, ZEB1 and *POLQ* expression are mutually exclusive in breast tumors. Secondly, ZEB1 inhibits *POLQ* transcription by directly binding to the *POLQ* promoter. Transcription inhibition and the resulting reduction of POLθ protein levels strongly impacts TMEJ activity. The use of a functional HPRT assay clearly demonstrated that ZEB1 limits TMEJ-associated genomic instability through the regulation of *POLQ* transcription (Prodhomme et al., 2021).

**FIGURE 2:**
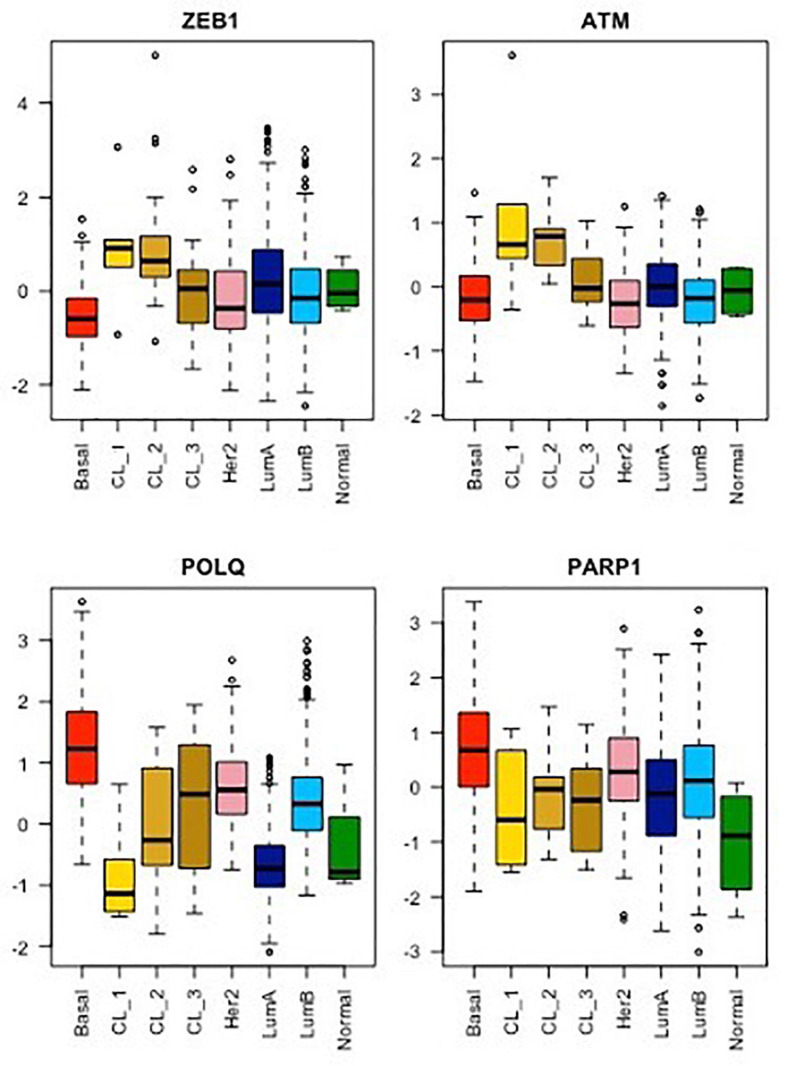
mRNA expression analysis of ZEB1, ATM, POLQ, and PARP1 for each breast cancer molecular subtype from the METABRIC cohort. As already shown, the CL1 subgroup shows the highest stemness and EMT phenotype, exemplify here by *ZEB1* expression, while CL2 and CL3 subgroups display an intermediate stemness and EMT phenotype compared to their relative luminal/basal counterparts and CL1 tumors (Pommier et al., 2020). The mRNA levels of *ATM*, a major player in DDR signaling, as well as of *POLQ* and *PARP1*, two major players in TMEJ, were analyzed for all breast cancer subtypes using the same method. Wilcoxon tests. Boxplot: center line, median; box limits, upper and lower quartiles; whiskers, minimum to maximum; all data points are shown.

This new piece of evidence showing the reduction of TMEJ activity by ZEB1 contributes to explaining the paucity of genomic aberrations displayed by ZEB1-expressing tumors. ZEB1 expression is a hallmark of claudin-low breast tumors (Morel et al., 2012; Fougner et al., 2019; Stemmler et al., 2019; Pommier et al., 2020) and ZEB1 counteracts the onset of oxidative stress in response to oncogene-induced replicative insults (Morel et al., 2017). Moreover, *POLQ* expression level is low in all CL tumors as compared to other breast cancer subtypes (Prodhomme et al., 2021). However, the characterization of three separate claudin-low subgroups, namely CL1, CL2 and CL3, with distinct transcriptomic, epigenetic, and genetic features led us to speculate on the correlation between the expression of TMEJ factors and ZEB1 levels in each subgroup (Pommier et al., 2020). Indeed, we showed here that *POLQ* expression is the lowest in CL1, displaying the highest level of ZEB1 while *POLQ* shows the highest expression in CL3, presenting the lowest level of ZEB1 (Figure 2). CL1 was shown to be enriched in stem-cell-related signatures (Pommier et al., 2020) with low proliferation activity. In contrast to CL1, the CL3 subgroup, containing the majority of BRCA-deficient tumors and showing lower levels of ZEB1, lower levels of ATM and higher levels of *POLQ*, displays stronger basal-related characteristics. In basal-like tumor subtypes with BRCA-mutations, ZEB1 expression may occur late in the oncogenic process depending on high *POLQ* expression in order to survive with the HR deficiency. In this case, even though ZEB1 downregulates *POLQ* expression, *POLQ* expression level remains higher than normal, yet slightly lower than in the basal-like subgroup. Interestingly, *ATM* expression follows *ZEB1* in all CL subtypes as anticipated with the already described mechanism of recruitment of the transcriptional coactivators p300/PCAF by ZEB1 to the *ATM* promoter (Zhang et al., 2018). Similarly, ATM-mediated stabilization of ZEB1 plays an important role for the enhanced accurate DNA repair ability by HR pathway of radioresistant tumor cells (Zhang et al., 2014). *POLQ* and *ATM* were firstly described in mice as synthetic semi-lethality. Indeed, Atm-/- Polq-/- double mutant mice showed marked developmental disadvantage (Shima et al., 2004). Moreover, the co-inhibition of *ATM* and *POLQ* enhanced the sensitivity to radiotherapy or chemotherapy (Goff et al., 2009; Pan et al., 2020). All these data suggested a unique role of Polq in maintaining genomic integrity, which is probably distinctive from the major HR pathway regulated by ATM as evidenced by the extensive evidence for synthetic lethality between HR and TMEJ. ZEB1, stabilized by ATM, probably then acts as an inhibitor of TMEJ to promote accurate HR DNA repair.

Moreover, downregulation of *POLQ* by ZEB1 was reported to foster micronuclei formation (Prodhomme et al., 2021). Indeed, it was shown in several organisms and under various conditions that POLθ prevents micronuclei formation, whereas the loss of POLθ expression leads to an increase in micronuclei (Shima et al., 2004; Goff et al., 2009; Yousefzadeh et al., 2014). These observations strengthened the notion that TMEJ is a full-fledged pathway, since the absence of TMEJ in claudin-low tumors leads to micronuclei originating most likely from unrepaired DNA damage (Prodhomme et al., 2021). However, in this specific breast tumor subtype, micronuclei abundance, generally considered to be a hallmark of genome instability (Jdey et al., 2017), is associated with low genomic instability (Morel et al., 2017). We postulated that one of the reasons explaining why micronuclei have no apparent incidence on claudin-low genome stability is that a small fraction of claudin-low cells with excessive micronuclei and/or unrepaired DNA damage would die. This hypothesis is illustrated in the analysis of neutral comet tail moments after the simultaneous deletion of TGFβ and TMEJ pathways, where an increase in DNA fragmentation is observed after cell irradiation (Liu et al., 2018). Consequently, an augmentation of unrepaired DNA may lead to cell death. Notably, radiosensitivity is highest when both TGFβ signaling and *POLQ* function are inactive (Liu et al., 2018). Further characterization of upcoming ZEB1-expressing tumor cells is needed to confirm this hypothesis, but one may suggest that ZEB1 and *POLQ* have opposite and complementary roles in the control of both the stability and integrity of breast cancer cell genomes. In normal cells, endogenous levels of POLθ and ZEB1 offer a compromise between the role of ZEB1 in protecting the stability of the genome and that of POLθ in protecting its integrity. In pathological conditions such as breast cancer, this balance is possibly disturbed due to high replicative stress, except in ZEB1-expressing cells, as formerly demonstrated (Morel et al., 2017). This dysregulation may contribute to *POLQ* dependence for survival, especially in a BRCA-deficient context.

## Discussion

Recent studies have underlined the role of EMT in DNA repair pathway choice and in particular, TMEJ activity in breast cancers. TMEJ protects from replication stress by preserving genomic integrity at the cost of mutations in most breast cancer subtypes, except in BRCA non-mutated claudin-low subtypes, in which the important contribution of ZEB1 as a protective actor in both early and late steps of tumor development has been demonstrated (Morel et al., 2017; Pommier et al., 2020; Prodhomme et al., 2021).

Uncovering the mechanisms of TMEJ regulation in cancer progression remains an ongoing task. We have shown that the EMT transcription factor, ZEB1 interacts directly with the *POLQ* promoter to control the expression of the *POLQ* gene and prevent TMEJ activity. However, the mechanisms underlying the upregulation of *POLQ* expression, in particular in BRCA-deficient cancers, are still unknown.

POLθ was recently identified as a potential target in the treatment of numerous breast tumors, especially BRCA-deficient tumors. ZEB1 could constitute an important biomarker to exclude BRCA non-mutated, claudin-low tumors from future therapy with POLθ inhibitors. Conversely, considering the repressive role of ZEB1 on TMEJ activity, the identification of a ZEB1 inhibitor could be used to systematically stimulate TMEJ and render those tumors more sensitive to POLθ inhibition.

## Author Contributions

MP, J-SH and AT contributed to original draft preparation and editing. All authors contributed to the conceptualization of the review, the research of the pertinent literature and writing.

## Conflict of Interest

The authors declare that the research was conducted in the absence of any commercial or financial relationships that could be construed as a potential conflict of interest.

## Publisher’s Note

All claims expressed in this article are solely those of the authors and do not necessarily represent those of their affiliated organizations, or those of the publisher, the editors and the reviewers. Any product that may be evaluated in this article, or claim that may be made by its manufacturer, is not guaranteed or endorsed by the publisher.

## References

[B1] Alexander J. L. Beagan K. Orr-Weaver T. L. McVey M. (2016). Multiple mechanisms contribute to double-strand break repair at rereplication forks in *Drosophila* follicle cells. *Proc. Natl. Acad. Sci. U.S.A.* 113 13809–13814. 10.1073/pnas.1617110113 27849606 PMC5137741

[B2] Allera-Moreau C. Rouquette I. Lepage B. Oumouhou N. Walschaerts M. Leconte E. (2012). DNA replication stress response involving PLK1, CDC6, POLQ, RAD51 and CLASPIN upregulation prognoses the outcome of early/mid-stage non-small cell lung cancer patients. *Oncogenesis* 1:e30. 10.1038/oncsis.2012.29 23552402 PMC3503291

[B3] Arana M. E. Seki M. Wood R. D. Rogozin I. B. Kunkel T. A. (2008). Low-fidelity DNA synthesis by human DNA polymerase theta. *Nucleic Acids Res.* 36 3847–3856. 10.1093/nar/gkn310 18503084 PMC2441791

[B4] Bartkova J. Hořejší Z. Koed K. Krämer A. Tort F. Zleger K. (2005). DNA damage response as a candidate anti-cancer barrier in early human tumorigenesis. *Nature* 434 864–870. 10.1038/nature03482 15829956

[B5] Bergoglio V. Boyer A. S. Walsh E. Naim V. Legube G. Lee M. Y. W. T. (2013). DNA synthesis by pol η promotes fragile site stability by preventing under-replicated DNA in mitosis. *J. Cell Biol.* 201 395–408. 10.1083/jcb.201207066 23609533 PMC3639397

[B6] Bertolin A. P. Hoffmann J. S. Gottifredi V. (2020). Under-replicated DNA: the byproduct of large genomes? *Cancers* 12:2764. 10.3390/cancers12102764 32992928 PMC7601121

[B7] Bianco J. N. Bergoglio V. Lin Y. L. Pillaire M. J. Schmitz A. L. Gilhodes J. (2019). Overexpression of claspin and timeless protects cancer cells from replication stress in a checkpoint-independent manner. *Nat. Commun.* 10:910. 10.1038/s41467-019-08886-8 30796221 PMC6385232

[B8] Brabletz T. Kalluri R. Nieto M. A. Weinberg R. A. (2018). EMT in cancer. *Nat. Rev. Cancer* 18 128–134. 10.1038/nrc.2017.118 29326430

[B9] Carvajal-Garcia J. Cho J. E. Carvajal-Garcia P. Feng W. Wood R. D. Sekelsky J. (2020). Mechanistic basis for microhomology identification and genome scarring by polymerase theta. *Proc. Natl. Acad. Sci. U.S.A.* 117 8476–8485. 10.1073/pnas.1921791117 32234782 PMC7165422

[B10] Carvajal-Garcia J. Crown K. N. Ramsden D. A. Sekelsky J. (2021). DNA polymerase theta suppresses mitotic crossing over. *PLoS Genet.* 17:e1009267. 10.1371/journal.pgen.1009267 33750946 PMC8016270

[B11] Ceccaldi R. Liu J. C. Amunugama R. Hajdu I. Primack B. Petalcorin M. I. R. (2015). Homologous-recombination-deficient tumours are dependent on Polθ-mediated repair. *Nature* 518 258–262. 10.1038/nature14184 25642963 PMC4415602

[B12] David L. Fernandez-Vidal A. Bertoli S. Grgurevic S. Lepage B. Deshaies D. (2016). CHK1 as a therapeutic target to bypass chemoresistance in AML. *Sci. Signal.* 9:ra90. 10.1126/scisignal.aac9704 27625304

[B13] Feng W. Simpson D. A. Carvajal-Garcia J. Price B. A. Kumar R. J. Mose L. E. (2019). Genetic determinants of cellular addiction to DNA polymerase theta. *Nat. Commun.* 10:4286. 10.1038/s41467-019-12234-1 31537809 PMC6753077

[B14] Fougner C. Bergholtz H. Kuiper R. Norum J. H. Sørlie T. (2019). Claudin-low-like mouse mammary tumors show distinct transcriptomic patterns uncoupled from genomic drivers. *Breast Cancer Res.* 21:85. 10.1186/s13058-019-1170-8 31366361 PMC6670237

[B15] Franchet C. Hoffmann J. S. (2020). When RAD52 allows mitosis to accept unscheduled dna synthesis. *Cancers* 12:26. 10.3390/cancers12010026 31861741 PMC7017103

[B16] Goff J. P. Shields D. S. Seki M. Choi S. Epperly M. W. Dixon T. (2009). Lack of DNA Polymerase θ (POLQ) radiosensitizes bone marrow stromal cells in vitro and increases reticulocyte micronuclei after total-body irradiation. *Radiat. Res.* 172 165–174. 10.1667/RR1598.1 19630521 PMC2742993

[B17] Gorgoulis V. Vassiliou L.-V. F. Karakaidos P. Zacharatos P. Kotsinas A. Liloglou T. (2005). Activation of the DNA damage checkpoint and genomic instability in human precancerous lesions. *Nature* 434 907–913. 10.1038/nature03485 15829965

[B18] Hanahan D. Weinberg R. A. (2011). Hallmarks of cancer: the next generation. *Cell* 144 646–674. 10.1016/j.cell.2011.02.013 21376230

[B19] Hwang T. Reh S. Dunbayev Y. Zhong Y. Takata Y. Shen J. (2020). Defining the mutation signatures of DNA polymerase θ in cancer genomes. *NAR Cancer* 2:zcaa017. 10.1093/narcan/zcaa017 32885167 PMC7454005

[B20] Jdey W. Thierry S. Popova T. Stern M. H. Dutreix M. (2017). Micronuclei frequency in tumors is a predictive biomarker for genetic instability and sensitivity to the DNA repair inhibitor AsiDNA. *Cancer Res.* 77 4207–4216. 10.1158/0008-5472.CAN-16-2693 28588010

[B21] Kamp J. A. van Schendel R. Dilweg I. W. Tijsterman M. (2020). BRCA1-associated structural variations are a consequence of polymerase theta-mediated end-joining. *Nat. Commun.* 11:3615. 10.1038/s41467-020-17455-3 32680986 PMC7368036

[B22] Koole W. Schendel R. Van Karambelas A. E. Heteren J. T. Van, Okihara K. L. (2014). A Polymerase theta-dependent repair pathway suppresses extensive genomic instability at endogenous G4 DNA sites. *Nat. Commun.* 5:3216. 10.1038/ncomms4216 24496117

[B23] Lemee F. Bergoglio V. Fernandez-Vidal A. Machado-Silva A. Pillaire M.-J. Bieth A. (2010). DNA polymerase up-regulation is associated with poor survival in breast cancer, perturbs DNA replication, and promotes genetic instability. *Proc. Natl. Acad. Sci. U.S.A.* 107 13390–13395. 10.1073/pnas.0910759107 20624954 PMC2922118

[B24] Liu Q. Ma L. Jones T. Palomero L. Pujana M. A. Martinez-Ruiz H. (2018). Subjugation of TGFb signaling by human papilloma virus in head and neck squamous cell carcinoma shifts DNA repair from homologous recombination to alternative end joining. *Clin. Cancer Res.* 24 6001–6014. 10.1158/1078-0432.CCR-18-1346 30087144

[B25] Liu Q. Palomero L. Moore J. Guix I. Espín R. Aytés A. (2021). Loss of TGFB signaling increases alternative end-joining DNA repair that sensitizes to genotoxic therapies across cancer types. *Sci. Transl. Med.* 13:eabc4465. 10.1126/scitranslmed.abc4465 33568520 PMC8208885

[B26] Macheret M. Halazonetis T. D. (2015). DNA replication stress as a hallmark of cancer. *Annu. Rev. Pathol.* 10 425–448. 10.1146/annurev-pathol-012414-040424 25621662

[B27] Maiorano D. El Etri J. Franchet C. Hoffmann J. S. (2021). Translesion synthesis or repair by specialized dna polymerases limits excessive genomic instability upon replication stress. *Int. J. Mol. Sci.* 22:3924. 10.3390/ijms22083924 33920223 PMC8069355

[B28] Mateos-Gomez P. A. Gong F. Nair N. Miller K. M. Lazzerini-Denchi E. Sfeir A. (2015). Mammalian polymerase θ promotes alternative NHEJ and suppresses recombination. *Nature* 518 254–257. 10.1038/nature14157 25642960 PMC4718306

[B29] Morel A.-P. Ginestier C. Pommier R. M. Cabaud O. Ruiz E. Wicinski J. (2017). A stemness-related ZEB1-MSRB3 axis governs cellular pliancy and breast cancer genome stability. *Nat. Med.* 23 568–578. 10.1038/nm.4323 28394329

[B30] Morel A.-P. P. Hinkal G. W. Thomas C. C. Fauvet F. F. Courtois-Cox S. S. Wierinckx A. (2012). EMT inducers catalyze malignant transformation of mammary epithelial cells and drive tumorigenesis towards claudin-low tumors in transgenic mice. *PLoS Genet.* 8:e1002723. 10.1371/journal.pgen.1002723 22654675 PMC3359981

[B31] Negrini S. Gorgoulis V. G. Halazonetis T. D. (2010). Genomic instability an evolving hallmark of cancer. *Nat. Rev. Mol. Cell Biol.* 11 220–228. 10.1038/nrm2858 20177397

[B32] Pan Y.-R. Wu C.-E. Yeh C.-N. (2020). ATM inhibitor suppresses gemcitabine-resistant btc growth in a polymerase θ deficiency-dependent manner. *Biomolecules* 10:1529. 10.3390/biom10111529 33182492 PMC7697425

[B33] Patel P. S. Algouneh A. Hakem R. (2021). Exploiting synthetic lethality to target BRCA1/2-deficient tumors: where we stand. *Oncogene* 40 3001–3014. 10.1038/s41388-021-01744-2 33716297

[B34] Pettitt S. J. Frankum J. R. Punta M. Lise S. Alexander J. Chen Y. (2020). Clinical brca1/2 reversion analysis identifies hotspot mutations and predicted neoantigens associated with therapy resistance. *Cancer Discov.* 10 1475–1488. 10.1158/2159-8290.CD-19-1485 32699032 PMC7611203

[B35] Pillaire M. J. Selves J. Gordien K. Gouraud P. A. Gentil C. Danjoux M. (2010). A DNA replication signature of progression and negative outcome in colorectal cancer. *Oncogene* 29 876–887. 10.1038/onc.2009.378 19901968

[B36] Pommier R. M. Sanlaville A. Tonon L. Kielbassa J. Thomas E. Ferrari A. (2020). Comprehensive characterization of claudin-low breast tumors reflects the impact of the cell-of-origin on cancer evolution. *Nat. Commun.* 11 1–12. 10.1038/s41467-020-17249-7 32647202 PMC7347884

[B37] Prodhomme M. K. Pommier R. M. Franchet C. Fauvet F. Bergoglio V. Brousset P. (2021). EMT transcription factor ZEB1 represses the mutagenic POLθ-mediated end-joining pathway in breast cancers. *Cancer Res.* 81 1595–1606. 10.1158/0008-5472.can-20-2626 33239429

[B38] Saldivar J. C. Cortez D. Cimprich K. A. (2017). The essential kinase ATR: ensuring faithful duplication of a challenging genome. *Nat. Rev. Mol. Cell Biol.* 18 622–636. 10.1038/nrm.2017.67 28811666 PMC5796526

[B39] Sansregret L. Swanton C. (2017). The role of aneuploidy in cancer evolution. *Cold Spring Harb. Perspect. Med.* 7:a028373. 10.1101/cshperspect.a028373 28049655 PMC5204330

[B40] Schimmel J. Kool H. van Schendel R. Tijsterman M. (2017). Mutational signatures of non-homologous and polymerase theta-mediated end-joining in embryonic stem cells. *EMBO J.* 36 3634–3649. 10.15252/embj.201796948 29079701 PMC5730883

[B41] Schimmel J. van Schendel R. den Dunnen J. T. Tijsterman M. (2019). Templated insertions: a smoking gun for polymerase theta-mediated end joining. *Trends Genet.* 35 632–644. 10.1016/j.tig.2019.06.001 31296341

[B42] Schrempf A. Slyskova J. Loizou J. I. (2021). Targeting the DNA repair enzyme polymerase θ in cancer therapy. *Trends Cancer* 7 98–111. 10.1016/j.trecan.2020.09.007 33109489

[B43] Seki M. Marini F. Wood R. D. (2003). POLQ (Pol θ), a DNA polymerase and DNA-dependent ATPase in human cells. *Nucleic Acids Res.* 31 6117–6126. 10.1093/nar/gkg814 14576298 PMC275456

[B44] Seki M. Wood R. D. (2008). DNA polymerase θ (POLQ) can extend from mismatches and from bases opposite a (6-4) photoproduct. *DNA Repair* 7 119–127. 10.1016/j.dnarep.2007.08.005 17920341 PMC2185714

[B45] Shima N. Munroe R. J. Schimenti J. C. (2004). The mouse genomic instability mutation chaos1 is an allele of polq that exhibits genetic interaction with atm. *Mol. Cell. Biol.* 24 10381–10389. 10.1128/MCB.24.23.10381-10389.2004 15542845 PMC529050

[B46] Shirakihara T. Saitoh M. Miyazono K. (2007). Differential regulation of epithelial and mesenchymal markers by δEF1 proteins in epithelial-mesenchymal transition induced by TGF-β. *Mol. Biol. Cell* 18 3533–3544. 10.1091/mbc.E07-03-0249 17615296 PMC1951739

[B47] Stemmler M. P. Eccles R. L. Brabletz S. Brabletz T. (2019). Non-redundant functions of EMT transcription factors. *Nat. Cell Biol.* 21 102–112. 10.1038/s41556-018-0196-y 30602760

[B48] Thyme S. B. Schier A. F. (2016). Polq-mediated end joining is essential for surviving dna double-strand breaks during early zebrafish development. *Cell Rep.* 15 707–714. 10.1016/j.celrep.2016.03.072 27149851 PMC5063659

[B49] Wang Z. Song Y. Li S. Kurian S. Xiang R. Chiba T. (2019). DNA polymerase θ (POLQ) is important for repair of DNA double-strand breaks caused by fork collapse. *J. Biol. Chem.* 294, 3909–3919. 10.1074/jbc.RA118.005188 30655289 PMC6422074

[B50] Wyatt D. W. Feng W. Conlin M. P. Yousefzadeh M. J. Roberts S. A. Mieczkowski P. (2016). Essential roles for polymerase θ-mediated end joining in the repair of chromosome breaks. *Mol. Cell* 63 662–673. 10.1016/j.molcel.2016.06.020 27453047 PMC4992412

[B51] Ye X. Weinberg R. A. (2015). Epithelial-mesenchymal plasticity: a central regulator of cancer progression. *Trends Cell Biol.* 25 675–686. 10.1016/j.tcb.2015.07.012 26437589 PMC4628843

[B52] Yousefzadeh M. J. Wyatt D. W. Takata K. Mu Y. Hensley S. C. Tomida J. (2014). Mechanism of suppression of chromosomal instability by DNA polymerase POLQ. *PLoS Genet.* 10:e1004654. 10.1371/journal.pgen.1004654 25275444 PMC4183433

[B53] Zahn K. E. Averill A. M. Aller P. Wood R. D. Doublié S. (2015). Human DNA polymerase θ grasps the primer terminus to mediate DNA repair. *Nat. Struct. Mol. Biol.* 22 304–311. 10.1038/nsmb.2993 25775267 PMC4385486

[B54] Zeman M. K. Cimprich K. A. (2014). Causes and consequences of replication stress. *Nat. Cell Biol.* 16 2–9. 10.1038/ncb2897 24366029 PMC4354890

[B55] Zhang P. Wei Y. Wang L. Debeb B. G. Yuan Y. Zhang J. (2014). ATM-mediated stabilization of ZEB1 promotes DNA damage response and radioresistance through CHK1. *Nat. Cell Biol.* 16 864–875. 10.1038/ncb3013 25086746 PMC4150825

[B56] Zhang X. Zhang Z. Zhang Q. Zhang Q. Sun P. Xiang R. (2018). ZEB1 confers chemotherapeutic resistance to breast cancer by activating ATM. *Cell Death Dis.* 9:57. 10.1038/s41419-017-0087-3 29352223 PMC5833408

